# Acarbose-metformin is more effective in glycemic variability control than repaglinide-metformin in T2DM patients inadequately controlled with metformin: a retrospective cohort study

**DOI:** 10.7717/peerj.9905

**Published:** 2020-10-02

**Authors:** Guoli Du, Wanrun Xie, Yinxia Su, Yao Ma, Xiaoming Gao, Sheng Jiang, Huazheng Liang

**Affiliations:** 1 Department of Endocrinology, The First Affiliated Hospital of Xinjiang Medical University, Urumuqi, Xinjiang Uygur Autonomous Region, China; 2 Health Management Center, The First Affiliated Hospital of Xinjiang Medical University, Urumuqi, Xinjiang Uygur Autonomous Region, China; 3 Department of Endocrinology, The Second Mercy Hospital of Xinjiang Uygur Autonomous Region, Urumuqi, Xinjiang Uygur Autonomous Region, China; 4 Department of Cardiology, The First Affiliated Hospital of Xinjiang Medical University, Urumuqi, Xinjiang Uygur Autonomous Region, China; 5 Baker IDI Heart and Diabetes Institute, Melbourne, VIC, Australia; 6 Department of Neurology, Translational Research Institute of Brain and Brain-like Intelligence, Shanghai Fourth People’s Hospital Affiliated toTongji University, Shanghai, China

**Keywords:** Diabetes mellitus, Metformin, Acarbose, Repaglinide, Glucose variability

## Abstract

**Background:**

Acarbose and repaglinide are widely used either by themselves or in combination with other medications. However, their efficacy in diabetes control has not been compared when used in combination with metformin.

**Methods:**

The present study aimed to compare their effects on glycemic variability (GV) control when taken with metformin for type 2 diabetes mellitus (T2DM) inadequately controlled with metformin alone. In this retrospective cohort study, T2DM patients who were treated with either acarbose-metformin or repaglinide-metformin combination were recruited. Either acarbose 100 mg or repaglinide 2 mg triple daily was taken for the subsequent 12 weeks in combination with metformin. Demographic data, biochemical data and 7-point glycemic self-monitoring conducted with capillary blood (SMBG) data were reviewed after one week and 12 weeks. The primary outcome including glucose control and changes in GV as well as other factors affecting GV and the incidence of hypoglycemia were also analyzed.

**Results:**

Of the 305 T2DM patients enrolled, data from 273 subjects, 136 in the acarbose-metformin group (M+A) and 137 in the repaglinide-metformin group (M+R) were analyzed. Both regimens improved glycemic control at 12 weeks post commencement of new medications. GV, expressed as the mean amplitude of plasma glycemic excursions (MAGE, 5.0 ± 2.6 vs. 2.8 ± 1.6 mmol/L, *p* < 0.001 in M+A; 5.1 ± 2.5 vs. 2.9 ± 1.3 mmol/L, *p* < 0.001 in M+R), standard deviation of blood glucose (SDBG, 3.6 ± 1.3 vs. 2.0 ± 0.9 mmol/L, *p* < 0.001 in M+A; 3.7 ± 1.3 vs. 2.4 ± 1.3 *p* < 0.001 in M+R), coefficient of variation of blood glucose (CVBG, (0.30 ± 0.09 vs. 0.21 ± 0.1, *p* < 0.001 in M+A; 0.31 ± 0.09 vs. 0.24 ± 0.12, *p* < 0.001 in M+R), postprandial amplitude of glycemic excursions (PPGE, 5.2 ± 2.6 vs. 2.8 ± 1.6 mmol/L, *p* < 0.001 in M+A; 5.3 ± 2.5 vs. 2.9 ± 1.3 mmol/L, *p* < 0.001 in M+R) or largest amplitude of glycemic excursions (LAGE, 9.8 ± 3.6 vs. 5.4 ± 2.4 mmol/L, *p* < 0.001 in M+A; 10.1 ± 3.4 vs. 6.3 ± 3.2 mmol/L, *p* < 0.001 in M+R) decreased significantly after the addition of acarbose or repaglinide (*p* < 0.05 respectively). Compared with repaglinide-metformin, acarbose-metformin was more effective in GV control at 12 weeks post commencement of new medications (*p* < 0.05). This study indicates that both acarbose-metformin and repaglinide-metformin combinations could effectively reduce GV and the acarbose-metformin combination seems to be more effective than the repaglinide-metformin combination. However, this conclusion should be confirmed by future large-scaled and more comprehensive studies due to the limitations of the present study.

## Introduction

Type 2 diabetes mellitus (T2DM) is the sixth leading cause of disability in 2015 and it brings considerable socioeconomic pressures to the individuals, families, and global health economy (Collaborators GBoDS, 2015; Seuring, Archangelidi & Suhrcke, 2015). T2DM needs intensive management of glucose as well lipid and blood pressure to delay the occurrence and development of complications (ADA, 2018; Chatterjee, Khunti & Davies, 2017). Glycemic variability (GV), an indicator of glucose fluctuations, is a glycosylated hemoglobin (HbA1c)-independent risk factor of poor prognosis for diabetic patients with complications (Ceriello & Ihnat, 2010). GV, the mean daily glucose, as well as pre-prandial and postprandial glucose values could predict cardiovascular diseases in diabetes (Kilpatrick, Rigby & Atkin, 2008). Muggeo et al., (1997) reported that GV was correlated to mortality due to all etiologies and due to cardiovascular diseases in elderly type 2 diabetic patients. Other reports showed that GV was associated with carotid intima-media thickness (CIMT) in T2DM (Esposito et al., 2008; Temelkova-Kurktschiev et al., 1999). It has been reported that increased GV poses an increased risk of mortality in critically ill patients (Krinsley, 2008). In this retrospective study, it was found that the mortality in the lowest (first) quartile of GV was 12.1%, and it increased by nearly 50%, 125%, and 212% in the second, third, and fourth quartile, respectively. Another study also found that GV, particularly if accompanied by severe hypoglycemia, could increase mortality of both diabetic patients and non-diabetic patients in critical settings (Finfer et al., 2009).

Regarding the management of diabetes, GV should be taking into consideration along with the HbA1c level (Ceriello & Ihnat, 2010; Chinese CSoEi, 2017). As an essential part of the comprehensive management of diabetes, glycemic control could be self-monitored by measuring capillary blood (SMBG) or interstitial glucose (Inchiostro, Candido & Cavalot, 2013). SMBG, as a cost-effective and convenient method of monitoring, is useful for diabetes monitoring, especially for testing the effectiveness of lifestyle-targeted and pharmacological managements, and increasing patients’ compliance (ADA, 2018; Inchiostro, Candido & Cavalot, 2013).

Both the American Diabetes Association (ADA) guideline (ADA, 2018) and the European Association for the Study of Diabetes (EASD)-ADA consensus (Davies et al., 2018) on managing hyperglycemia in T2DM recommend metformin as the initial hypoglycemic medicine for patients with T2DM. As a foundation therapy for patients with T2DM, metformin has been widely used as the initial drug choice because of its efficacy, safety, low cost, and weight neutrality. Metformin is also efficacious when used in combination with other glucose-lowering medications for patients with T2DM inadequately controlled with metformin alone (Derosa et al., 2009; Lin et al., 2011). Asians, particularly Chinese, consume more carbohydrate-rich food, and usually have higher risk of uncontrolled postprandial hyperglycemia (PPHG) than caucasians. Acarbose competitively binds to *α*-glucosidase and inhibits the breakdown of carbohydrates. When used either alone or in combination with other glucose-lowering medications, it may reduce PPHG and improve GV in patients with T2DM (Weng et al., 2015). Repaglinide is a hypoglycemic drug that promotes insulin secretion and has the characteristics of quick start, short duration, and rapid metabolism. It has a low risk of hypoglycemia and great effect on postprandial hyperglycemia (Derosa et al., 2009; Fang et al., 2014; Moses et al., 1999; Omori et al., 2018). Combined use of acarbose or repaglinide with metformin or other medications will be needed if glycemic goals are not met. However, little research has been done to compare the efficacy of acarbose or repaglinide in controlling glucose variability when they are used individually in combination with metformin. Therefore, this study aimed to answer this question by retrospectively reviewing their efficacy using the SMBG method.

## Material and Methods

### Subjects

In the present retrospective study, participants were all patients managed by endocrinologists in our hospital. They received either acarbose-metformin or repaglinide-metformin after failing to respond to metformin only for at least 3 months. These patients were divided into two groups based on the medications they took. The selection criteria were: T2DM, ≥18 years of age, managed with metformin alone for at least 3 months with a level of HbA1c ≥7.0% and later on were prescribed with either acarbose-metformin or repaglinide-metformin due to the failure to respond to metformin alone. Patients’ data were excluded if they had received insulin or weight reduction drugs, they had impaired renal [calculated eGFR <45 ml/min/1.73 m^2^] or liver function, concomitant hemoglobinopathy or chronic anemia due to various etiologies, pregnant, lactating, or child-breeding females, or presence of cancer, or presence of diabetic ketoacidosis during the wash out period. This study was carried out by complying with the recommendations of ‘Guidelines of Human Research, Human Research Ethics Committee of the First Affiliated Hospital of Xinjiang Medical University’. Written informed consent was obtained from all participants recruited. The study protocol was approved by the Human Research Ethics Committee of the First Affiliated Hospital of Xinjiang Medical University (K202001-27).

### Drug administration

Participants received either acarbose 50 mg or repaglinide 1 mg three times a day in addition to metformin. Their doses were force titrated to 100 mg and 2 mg three times a day, respectively. These participants had been treated with the same medications and dosages for 12 or more weeks until the end of the study. Participants who had not been given the above regimens or/and whose medicine had not been forced titrated were not collected in this study.

### Anthropometric evaluation

Height, weight and blood pressure were reviewed from the record of each subject. The body mass index (BMI) was calculated using the formula: weight (kg)/height (m^2^) (Du et al., 2016). The mean value of the two blood pressure measurements was used for analysis. Hypertension was defined if the systolic pressure (SBP) was equal to or higher than 140 mmHg or /and the diastolic pressure (DBP) was equal to or higher than 90 mmHg or self-reported use of antihypertensive medications irrespective of measured blood pressure (Du et al., 2016).

### Biochemical assays

Levels of fasting blood glucose, HbA1c, total cholesterol (TC), triglyceride (TG), low-density lipoprotein cholesterol (LDL-c), and high-density lipoprotein cholesterol (HDL-c) were tested in the central laboratory of the First Affiliated Hospital of Xinjiang Medical University and they were available in the medical records of our participants.

### Glucose variability parameters

Blood glucose levels were measured for two consecutive days before each meal and 120 min after them, and at bedtime. MAGE (mean amplitude of glycemic excursions) was intended to evaluate the instability of blood glucose by assessing the mean of differences between consecutive increases or decreases surpassing 1 standard deviation (SD) around the mean of 24 h values of blood glucose. The SD of blood glucose (SDBG) was calculated based on the mean and SD of blood glucose measured at each visit. Coefficient of variation of blood glucose (CVBG) was calculated by dividing the SDBG using the mean blood glucose × 100%. The postprandial glucose excursion (PPGE) referred to the mean of differences between pre-prandial glucose values and postprandial (within 2 h) glucose values, whereas the largest amplitude of glycemic excursion (LAGE) referred to the maximum glucose level minus the minimum glucose level on the same day (Li et al., 2013).

These GV parameters were tested 1 week and 12 weeks post commencement of new medications. If not given, we would calculate these GV parameters according to those 7-point blood glucose. The subjects were divided into quintiles based on MAGE. We had defined the possible risk factors of MAGE as following: Age (≥60 years), ethnicity (Uygur vs. Han Chinese), education level (undergraduate or above), body mass index (≥28 kg/m^2^), history of cardiovascular diseases (Yes), diabetes duration (≥5 years), levels of 24-hour urine proteins (≥0.15 g), triglyceride (≥1.71 mmol/L), cholesterol (≥5.20 mmol/L), high density lipoprotein cholesterol (<1.04 mmol/L), low density lipoprotein cholesterol (≥3.38 mmol/L), and regiment of acarbose-metformin combination.

### Hypoglycemia

Hypoglycemia, as a side-effect of diabetic medications, was usually recorded in the medical records of diabetic patients. Both hypoglycemia symptoms and levels of blood glucose (SMBG readings <3.9) immediately measured after the onset of symptoms were searched in the record to confirm the presence of hypoglycemia. Severe hypoglycaemia was defined as SMBG readings ≤ 2.5 mmol/L.

### Statistical analysis

Statistical analysis was performed using SPSS 21.0. Data were presented as mean  ± SD for quantitative variables and assessed using an independent Student’s t test when data were normally distributed; otherwise, non-parametric data were compared using the Mann–Whitney/ Wilcoxon test. Chi-square test was used to analyze the differences between categorical variables. Changes in GV were divided into 5 quintiles from the smallest (quintile 1) to the largest (quintile 5). Quintile 3 was set as the reference and correlation between GV risk factors and other quintiles was analyzed. The multinomial logistic regression analysis was applied to test the confounding factors of GV. *P* < 0.05 denoted statistically significant difference.

## Results

### Patient demographics

**Figure 1:**
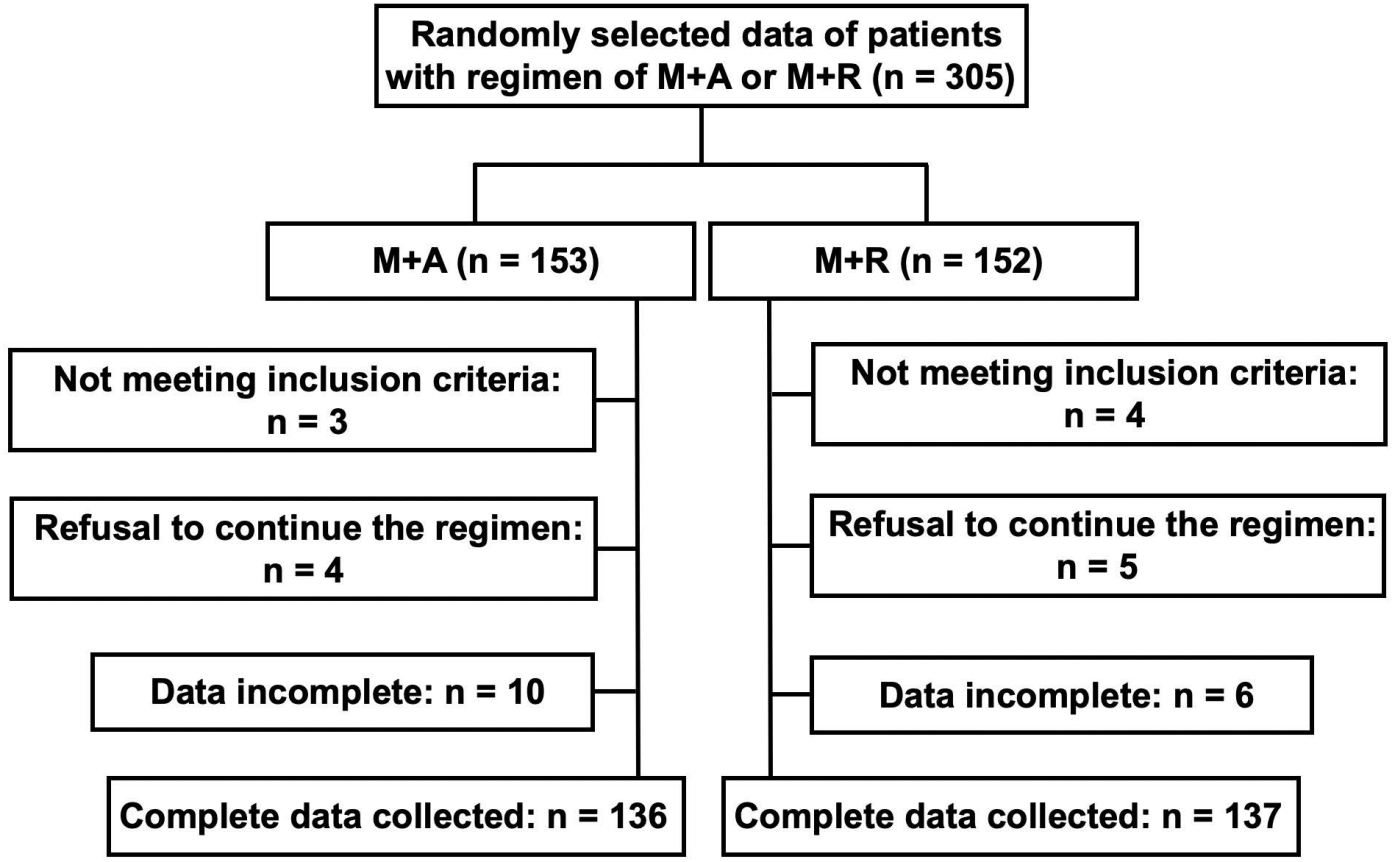
Flow chart for patient selection. M+A: Regime of acarbose-metformin combination; M+R: Regime of repaglinide-metformin combination.

The baseline information of the participants was listed in Table 1. Among 305 T2DM patients recruited, 32 were excluded due to incomplete information and data from 273 participants, including 136 treated with acarbose-metformin and 137 treated with repaglinide-metformin, were analyzed (Fig. 1). It could be seen that there was no significant difference (*p* > 0.05) in general clinical data such as gender, ethnicity, and educational background. There was no significant difference in medical histories of hypertension, cardiovascular diseases between these groups (*p* > 0.05, respectively). Also, no significant difference was observed in fasting plasma glucose (FPG), postprandial 2-hour plasma glucose (2hPG), BMI, HbA1c, 24-hour urinary protein, and lipid profiles between these groups (*p* > 0.05 respectively). Regarding the dose of metformin, there was no significant difference in the daily dose between these two groups.

**Table 1:** General characteristics of the participants.

	M+A (*n* = 136)	M+R (*n* = 137)	*p*-value
Gender, male	90 (66)	87 (64)	0.645
Age (years)	55.0 ± 11.5	55.3 ± 9.4	0.823
Ethnicity (Han, n/%)	100 (74)	100 (73)	0.921
Education level (undergraduate or above, n/%)	26 (19)	32 (23)	0.394
Cardiovascular disease (n/%)	94 (69)	92 (67)	0.729
Diabetes duration (years)	8.6 ± 6.5	9.4 ± 6.3	0.329
Body mass index (kg/m^2^)	26.1 ± 3.8	25.7 ± 3.1	0.261
HbA1c (%)	9.0 ± 1.8	9.1 ± 2.0	0.308
24 h urine protein (g)	0.1 ± 0.4	0.2 ± 0.7	0.565
Triglyceride (mmol/L)	2.4 ± 2.4	2.4 ± 2.2	0.968
Cholesterol (mmol/L)	4.4 ± 1.2	4.3 ± 1.2	0.708
High density lipoprotein cholesterol (mmol/L)	1.1 ± 0.3	1.2 ± 0.8	0.435
Low density lipoprotein cholesterol (mmol/L)	2.9 ± 0.9	2.8 ± 1.0	0.528
Metformin daily dose (g)	1.43 ± 0.37	1.42 ± 0.47	0.940

**Notes.**

Data are expressed as number (%) or mean SD. M+A: Regime of acarbose-metformin combination; M+R: Regime of repaglinide-metformin combination; *p* < 0.05 was considered to be significantly different.

### Glucose lowering effect and GV analysis

**Figure 2:**
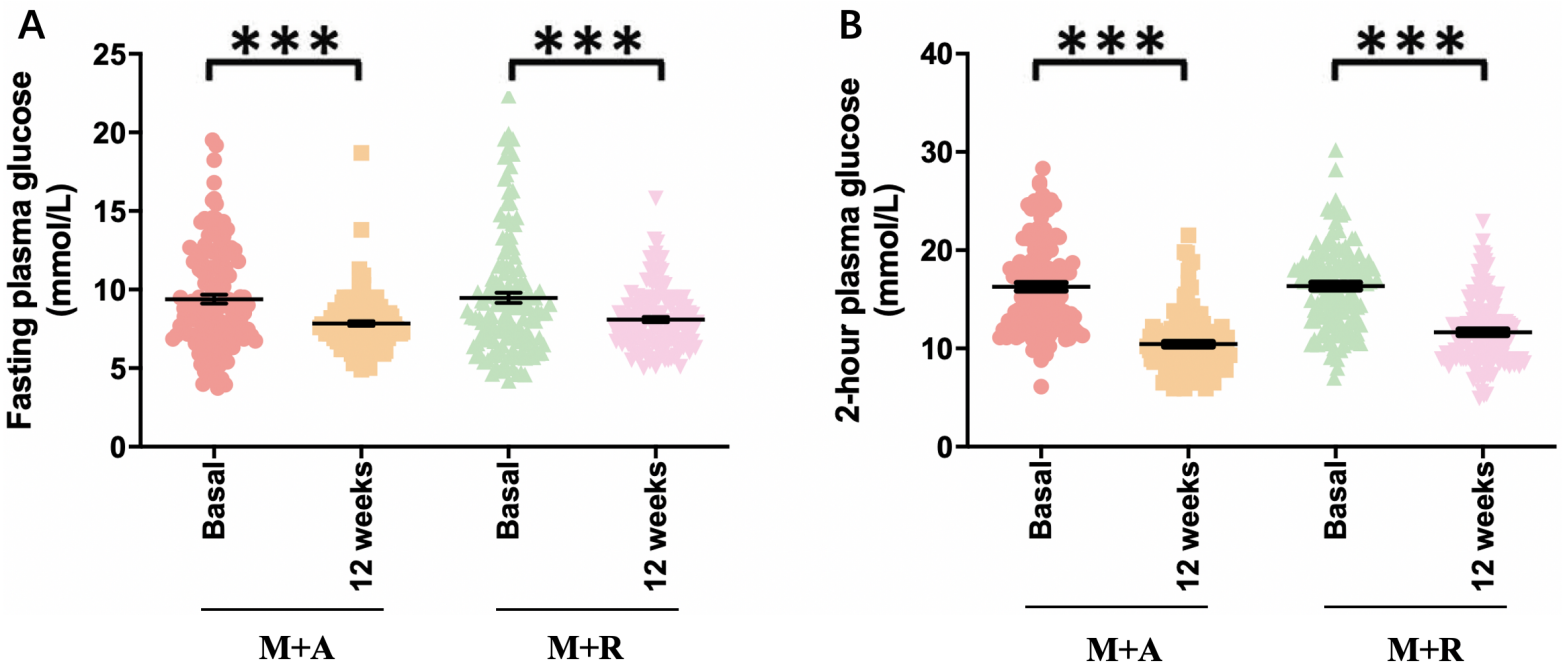
Hypoglycemia effect of both regimens. (A) HbA1c: glycosylated hemoglobin; (B) FPG: fasting plasma glucose; (C) 2hPG: 2-hour plasma glucose; *p*^∗∗∗^ < 0.001.

At the end of 12 weeks management with combined therapies (acarbose and repaglinide forced titrated to 100 mg and 2 mg three times a day), data of patients were collected. FPG decreased markedly in both groups at the time point of one week (*p* < 0.001, respectively, Table S1) and there was no significant difference in the reduction between the acarbose-metformin combination group and the repaglinide-metformin combination group (7.5 ± 1.8 vs. 7.1 ± 1.3 mmol/L, *p* = 0.099, Table S1). Glucose variability parameters including MAGE, SDBG, PPGE, and LAGE decreased significantly in both groups and the acarbose-metformin combination was more remarkable than the repaglinide-metformin group (*p* < 0.001 respectively, Table S1). FPG (9.4 ± 3.3 vs. 7.5 ± 1.8, *p* < 0.001) and glucose variability parameters also decreased markedly at the end of 12 weeks in both groups (*p* < 0.001, respectively, Fig. 2). Both regimens improved glycemic control at 12 weeks post commencement of new medications. GV, expressed as MAGE (5.0 ± 2.6 vs. 2.8 ± 1.6 mmol/L, *p* < 0.001 in M+A; 5.1 ± 2.5 vs. 2.9 ± 1.3 mmol/L, *p* < 0.001 in M+R), SDBG (3.6 ± 1.3 vs. 2.0 ± 0.9 mmol/L, *p* < 0.001 in M+A; 3.7 ± 1.3 vs. 2.4 ± 1.3, *p* < 0.001 in M+R), CVBG (0.30 ± 0.09 vs. 0.21 ± 0.1, *p* < 0.001 in M+A; 0.31 ± 0.09 vs. 0.24 ± 0.12, *p* < 0.001 in M+R ), PPGE (5.2 ± 2.6 vs. 2.8 ± 1.6 mmol/L, *p* < 0.001 in M+A; 5.3 ± 2.5 vs. 2.9 ± 1.3 mmol/L, *p* < 0.001 in M+R) or LAGE (9.8 ± 3.6 vs. 5.4 ± 2.4 mmol/L, *p* < 0.001 in M+A; 10.1 ± 3.4 vs. 6.3 ± 3.2 mmol/L, *p* < 0.001 in M+R) decreased significantly after the addition of acarbose or repaglinide (*p* < 0.05 respectively). Compared with repaglinide-metformin, acarbose-metformin was more effective in GV control at 12 weeks post commencement of new medications (*p* < 0.05 respectively), demonstrated by the smaller MAGE, SDBG, CVBG, PPGE, and LAGE in the acarbose-metformin combination group than in the repaglinide-metformin combination group (*p* < 0.05 respectively, Table 2). The acarbose-metformin combination was more effective in GV control as shown by the smaller MAGE, SDBG, PPGE and LAGE in the acarbose-metformin combination group (*p* < 0.05 respectively, Table 2).

**Table 2:** Glucose variability in different regiments at the time point of 12 weeks

	M+A (*n* = 136)				M+R (*n* = 137)				p^x^-value
	Basal	12 weeks	Change (%)	*p*-value	Basal	12 weeks	Change (%)	*p*-value	
MAGE (mmol/L)	5.0 ± 2.6	2.8 ± 1.6	44.7	<0.001	5.1 ± 2.5	2.9 ± 1.3	43.3	<0.001	0.046
SDBG (mmol/L)	3.6 ± 1.3	2.0 ± 0.9	44.5	<0.001	3.7 ± 1.3	2.4 ± 1.3	35.1	<0.001	0.008
PPGE (mmol/L)	5.2 ± 2.6	2.8 ± 1.6	46.1	<0.001	5.3 ± 2.5	2.9 ± 1.3	45.3	<0.001	0.010
LAGE (mmol/L)	9.8 ± 3.6	5.4 ± 2.4	44.9	<0.001	10.1 ± 3.4	6.3 ± 3.2	37.6	<0.001	0.001

**Notes.**

Data are expressed as mean ± SD. M+A, regime of acarbose-metformin combination; M+R, regime of repaglinide-metformin combination; MAGE, mean amplitude of plasma glycemic excursions; SDBG, standard deviation of blood glucose; PPGE, postprandial amplitude of glycemic excursions; LAGE, largest amplitude of glycemic excursions. *p* < 0.05 was considered to be significantly different; P^x^: Comparison between both groups at the time point of 12 weeks.

### Hypoglycemia incidence analysis

No hypoglycemia led to drug discontinuation in either the acarbose-metformin combination group or the repaglinide-metformin combination group. None of the 273 patients experienced severe hypoglycemia (defined as severely impaired consciousness caused by hypoglycemia requiring assistance of others and hospitalization). No significant difference in the hypoglycemic incidence was found between the acarbose-metformin combination group and the repaglinide-metformin combination group (*p* > 0.05). In the acarbose-metformin combination group, one patient had hypoglycemic symptoms with fasting blood glucose less than 3.9 mmol/L and one patient had blood glucose less than 2.5 mmol/L. In the repaglinide-metformin combination group, one patient experienced hypoglycemic symptoms with blood glucose less than 3.9 mmol/L and no patients’ blood glucose was less than 2.5 mmol/L (Table 3). Another well-known side effect of metformin and alpha-glucosidase inhibitor is gastric intolerance (GI).

**Table 3:** Incidence of hypoglycemia.

	M+A	M+R	*p*-value
2.5 mmol/L <Pre-prandial glucose ≤ 3.9 mmol/L	1/136 (0.7)	1/137 (0.7)	0.993
2.5 mmol/L <Postprandial glucose ≤ 3.9 mmol/L	0/136 (0)	0/137 (0)	
2.5 mmol/L <Bedtime glucose ≤ 3.9 mmol/L	0/136 (0)	0/137 (0)	
Pre-prandial glucose ≤ 2.5 mmol/L)	1/136 (0.7)	0/137 (0)	0.315
Postprandial glucose ≤ 2.5 mmol/L	0/136 (0)	0/137 (0)	
Bedtime glucose ≤ 2.5 mmol/L	0/136 (0)	0/137 (0)	
Total, *n* (%)	2/136(1.5)	1/137 (0.7)	0.557

**Notes.**

Data are expressed as *n* (%). M+A, regime of acarbose-metformin in combination; M+R, regime of repaglinide-metformin combination; *p* < 0.05 was considered to be significantly different.

### Factors that influence GV

Multinomial logistic regression analysis identified regimen of acarbose-metformin combination as an independent determinant of GV (employing MAGE as a dependent variable) over the 12 weeks study period. The acarbose-metformin combination regimen was likely to decrease GV after adjusting gender, age, ethnicity, education level, BMI, and lipid profiles. In the present study, the odds ratio (OR) of MAGE in the third quintile was set as the reference, which equalled to 1, the ORs of MAGE in the first, second, fourth, and fifth quintile were 0.65 (95% CI: 0.29 to 1.42), 0.99 (95% CI: 0.44 to 2.21), 0.55 (95% CI: 0.25 to 1.23), and 0.25 (95% CI: 0.11 to 0.58), respectively (*p* = 0.006, Table 4).

**Table 4:** Pooled results for the association of changes in MAGE after 12 weeks with possible risk factors of glycemic variability (reference: population prescribed regiment of repaglinide-metformin combination).

Variable	Quintile of Changes of MAGE					*p*-value
	1 (0.07–1.63)	2 (−2.37)	3 (−3.07)	4 (−3.87)	5 (−7.13)	
Age (≥60 years)	0.59 (0.08–4.22)	5.32 (0.66–43.07)	1.00	3.42 (0.42–28.24)	2.64 (0.33–21.37)	0.059
Ethnicity (Uygur vs. Han Chinese)	0.49 (0.19–1.27)	1.01 (0.41–2.44)	1.00	0.43 (0.16–1.15)	0.57 (0.22–1.49)	0.237
Education level (undergraduate or above)	1.55 (0.57–4.19)	0.57 (0.22–1.48)	1.00	1.38 (0.50–3.87)	1.40 (0.51–3.85)	0.273
Body mass index (≥28 kg/m^2^)	1.93 (0.54–6.91)	2.84 (0.86–9.37)	1.00	2.58 (0.76–8.69)	2.00 (0.57–6.97)	0.557
History of Cardiovascular disease (Yes)	0.99 (0.40–2.42)	1.08 (0.42–2.78)	1.00	0.60 (0.25–1.47)	0.52 (0.21–1.27)	0.363
Diabetes duration (≥5 years)	1.46 (0.57–3.72)	2.23 (0.88–5.70)	1.00	1.71 (0.65–4.45)	2.16 (0.83–5.64)	0.428
24 h urine protein (≥0.15 g)	0.53 (0.16–1.70)	1.48 (0.53–4.11)	1.00	1.61 (0.58–4.49)	0.94 (0.30–2.96)	0.288
Triglyceride (≥1.71 mmol/L)	0.48 (0.20–1.13)	0.69 (0.29–1.68)	1.00	0.55 (0.23–1.34)	1.24 (0.50–3.06)	0.161
Cholesterol (≥5.20 mmol/L)	0.91 (0.22–3.76)	0.86 (0.19–3.84)	1.00	1.78 (0.35–8.99)	0.45 (0.09–2.35)	0.631
High density lipoprotein cholesterol (<1.04 mmol/L)	0.67 (0.28–1.59)	1.72 (0.73–4.06)	1.00	1.58 (0.67–3.74)	1.09 (0.46–2.62)	0.209
Low density lipoprotein cholesterol (≥3.38 mmol/L)	0.52 (0.14–1.86)	0.61 (0.15–2.40)	1.00	1.99 (0.43–9.18)	1.12 (0.27–4.60)	0.345
Regiment of acarbose-metformin combination	0.65 (0.29–1.42)	0.99 (0.44–2.21)	1.00	0.55 (0.25–1.23)	0.25 (0.11–0.58)	0.006

**Notes.**

Data are expressed as Odds Ratio (95% CI). *p* < 0.05 was considered to be significantly different. MAGE, mean amplitude of plasma glycemic excursions.

## Discussion

The present study found that both acarbose-metformin and repaglinide-metformin combinations improved glycemic control and effectively reduced GV in type 2 diabetes mellitus patients inadequately controlled with metformin alone. The acarbose-metformin combination was more effective in reducing GV than the repaglinide-metformin combination.

Metformin is the first-line oral medication for lowering blood glucose of T2DM patients (Sanchez-Rangel & Inzucchi, 2017; Scarpello & Howlett, 2008). It has been recommended by the majority of guideline committees for type 2 diabetic patients to take if they are unable to control the level of blood glucose to the targets despite completing lifestyle modifications (ADA, 2018; Davies et al., 2018).

GV may lead to complications associated with fluctuations of blood glucose. It is, therefore, the goal of diabetes management to minimize blood glucose fluctuation from one extreme to the other, and to decrease mortality and disability associated with diabetes mellitus (Cavalot et al., 2006; Ceriello & Ihnat, 2010; Muggeo et al., 2000; Nalysnyk, Hernandez-Medina & Krishnarajah, 2010; Suh & Kim, 2015). The immediate response to glucose fluctuation is endothelial dysfunction demonstrated by reduced nitric oxide availability, increased non-enzymatic glycation or oxidative stress, contributing to vascular complications (Wang et al., 2011). GV may not be noticeable for some patients who had ‘glucose control to target level’, such as normal levels of blood glucose and low levels of HbA1c.

It is expected that available regimens of combined medications are able to control the level of blood glucose and consequently are able to decrease GV and associated complications. For example, acarbose, a drug that targets postprandial hyperglycemia, might decrease glycemic excursions and oxidative stress. As a result, it improves endothelial function of patients with T2DM or impaired glucose tolerance (Li et al., 2010; Shimabukuro et al., 2006). It has been reported that the combination of acarbose and metformin was more efficient in decreasing GV than the combination of glibenclamide and metformin ^[19]^, demonstrating the efficacy of acarbose in GV control.

Repaglinide is another medication which can improve GV by promoting insulin release from the pancreas with a low risk of developing hypoglycemia (Hasslacher, 2003; Jovanovic et al., 2000). It has been reported that elderly patients with T2DM had attenuated glucose fluctuation after switching from sulfonylurea to repaglinide (Lin et al., 2011; Omori et al., 2018). In the present study, we found that acarbose add-on more remarkably reduced GV than repaglinide add-on although both of them may improve GV. A study reported that acarbose-glipizide controlled-release tablets were more effective in reducing intra-day and day-to-day GV than sole glipizide controlled-release tablets (Bao et al., 2010). Another study found that acarbose and nateglinide were similar in glycemic control, but acarbose seemed to be better than nateglinide in controlling early (30 and 60 min) postprandial glucose excursions (Li et al., 2013). Therefore, the acarbose-metformin combination might be a good alternative add-on medication for those who do not benefit from metformin monotherapy in GV control. Our comparison of the hypoglycemic efficacies between acarbose-metformin and repaglinide-metformin combinations will guide the selection of suitable medications.

The prevalence of cardiovascular diseases was high (68%) among patients in the present study, and higher than the global level. Diabetes mellitus is known as a risk factor of cardiovascular diseases (Chatterjee, Khunti & Davies, 2017). As the major complication of T2DM, cardiovascular diseases are the most common cause of death of diabetic patients. Compared with people who do not have cardiovascular diseases, T2DM increases the risk of death by three to four times (Jeremiah Stamler et al., 1993). A recent meta-analysis showed that approximately 32.2% of patients with T2DM had cardiovascular diseases and this accounted for 50.3% of all deaths of this population (Einarson et al., 2018). In other randomized controlled trials (RCTs), the prevalence of CVD ranged from 30% to 80% (UK Prospective Diabetes Study, 1998; Marian Sue, Hussain & Korytkowski, 2018; Marso et al., 2016; Zinman et al., 2015). The high prevalence of CVD in our study might be attributed to data selection bias. For example, most participants were selected from the hospitalized patients, and these patients may have more CVD complications. Their blood glucose was not well managed with metformin alone due to the complexity of their conditions. This constantly high level of blood glucose may also contribute to the high prevalence of CVD. Finally, these patients tended to be older (>55 years) and had a longer duration (9 years) of diabetes than those who do not have CVD. This might be another reason for the high prevalence of CVD.

This study has a number of limitations. Firstly, 7-point SMBG might fail to monitor episodes of possible glycemic excursions during a day although it is as valid as CGM for monitoring GV (Service, 2013; Wang et al., 2015). For patients who monitor their fingertip blood glucose at home, different glucometers they use might have discrete accuracy although the systematic bias of glucose concentration has been minimized to 0.5 mmol/L among different glucometers and laboratory blood tests. Secondly, the influence of diets and lifestyles could not be excluded because patients may have different types and amounts of food as well as exercises. Therefore, GV could be influenced by many factors. Thirdly, the therapeutic effect of diabetic medications on GV might be influenced by insulin resistance and the function of beta cells. However, few patients in the present study had tested insulin resistance or the function of beta cells. Therefore, it is impossible to test the impact of insulin resistance and beta cell function on GV control. Future studies should include this important information. Finally, patients who have been prescribed both acarbose and repaglinide were excluded in the present study, only those who need to switch from metformin alone to the combined approach were recruited. Therefore, clinicians’ preference of medications can not be excluded. This can be a source of allocation bias.

## Conclusions

The present study demonstrated that acarbose-metformin combination and repaglinide-metformin combination can effectively reduce glycemic variability and the acarbose-metformin combination is more effective than the repaglinide-metformin combination in glycemic variability control. However, larger scaled and more comprehensive studies are required to confirm our findings due to the limitations of the present study.

##  Supplemental Information

10.7717/peerj.9905/supp-1 Supplemental Information 1 Original results collected from participants Each row represents a participant and each column represents a parameter about the participants.

10.7717/peerj.9905/supp-2 Table S1 Flow chart from patient randomization to completion Recruitment process of participants.

## Additional Information and Declarations

### Data Availability

The following information was supplied regarding data availability: All information about the participants and their diabetes-related parameters are available in the Supplemental File.
