# Supplementary material for: Acarbose-metformin is more effective in glycemic variability control than repaglinide-metformin in T2DM patients inadequately controlled with metformin: a retrospective cohort study
Source: PeerJ. 2020 Oct 2;8:e9905. doi: 10.7717/peerj.9905 (PMC7537614; doi:10.7717/peerj.9905)
Supplement: Table S1 — Recruitment process of participants. [file peerj-08-9905-s002.doc]

**Supplementary Table 1. Glucose variability in different regimes at the time point of one week**

|  | M+A (n = 136) | | |  | M+R (n = 137) | | |  | p^§^-value |
| --- | --- | --- | --- | --- | --- | --- | --- | --- | --- |
|  | Basal | One week | p-value |  | Basal | One week | p-value |  |  |
| FPG (mmol/L) | 9.4 ± 3.3 | 7.5 ± 1.8 | < 0.001 |  | 9.5 ± 3.8 | 7.1 ± 1.3 | < 0.001 |  | 0.099 |
| 2hPG (mmol/L) | 16.3 ± 4.5 | 10.7 ± 2.8 | < 0.001 |  | 16.4 ± 4.2 | 11.0 ± 2.7 | < 0.001 |  | 0.307 |
| MAGE (mmol/L) | 5.0 ± 2.6 | 2.5 ± 1.2 | < 0.001 |  | 5.1 ± 2.5 | 3.0 ± 1.6 | < 0.001 |  | 0.002 |
| SDBG (mmol/L) | 3.6 ± 1.3 | 3.0 ± 0.3 | < 0.001 |  | 3.7 ± 1.3 | 3.3 ± 0.4 | < 0.001 |  | < 0.001 |
| CVBG  (mmol/L) | 0.30±0.09 | 0.34±0.03 | ＜0.001 |  | 0.31±0.09 | 0.36±0.06 | ＜0.001 |  | ＜0.001 |
| PPGE (mmol/L) | 5.2 ± 2.6 | 2.5 ± 1.2 | < 0.001 |  | 5.3 ± 2.5 | 3.0 ± 1.7 | < 0.001 |  | < 0.001 |
| LAGE (mmol/L) | 9.8 ± 3.6 | 5.6 ± 2.1 | < 0.001 |  | 10.1 ± 3.4 | 6.9 ± 2.9 | < 0.001 |  | < 0.001 |

Data are expressed as mean ± SD. M+A – acarbose-metformin combination; M+R – regime of repaglinide-metformin combination; FPG – fasting plasma glucose; 2hPG – 2-hour plasma glucose; MAGE – mean amplitude of plasma glycemic excursions; SDBG – standard deviation of blood glucose; PPGE – postprandial amplitude of glycemic excursions; LAGE – largest amplitude of glycemic excursions. p < 0.05 was considered to be significantly different; *P^§^*: Comparison between both groups at the time point of one week.
